# Immature defense mechanisms and suicide attempts in borderline personality organization: a clinical sample study

**DOI:** 10.3389/fpsyg.2025.1632246

**Published:** 2025-09-03

**Authors:** Ardil Bayram Şahin, Mourat Giousouf Chousein, Doruk Uludağ, M. Bilgin Saydam

**Affiliations:** ^1^Research Center for Translational Medicine (KUTTAM), Koç University, Istanbul, Türkiye; ^2^Department of Psychiatry, Istanbul Faculty of Medicine, Istanbul University, Istanbul, Türkiye; ^3^Department of Psychiatry, Faculty of Medicine, Uskudar University, Istanbul, Türkiye

**Keywords:** borderline personality organization, suicide, defense mechanism, immature defense mechanism, projection, acting out, splitting

## Abstract

**Objective:**

This study aimed to investigate the relationship between defense mechanisms and suicide attempts in individuals with borderline personality organization (BPO), considering the high prevalence of suicide attempts in this population.

**Methods:**

A total of 80 participants (71.25% female) who met all inclusion criteria and had complete data were included in the analyses. They were recruited from the outpatient psychotherapy unit of a university hospital. All participants participated in clinical interviews and completed standardized psychometric scales, including the Borderline Personality Inventory (BPI), Defense Style Questionnaire, and Symptom Checklist-90 Revised (SCL-90-R). Participants were classified two group based on their history of suicide attempts. Statistical analyses, including group comparisons, correlation analyses, and regression models, were used to explore the relationship between defense mechanisms and suicide attempts among individuals with BPO.

**Results:**

Of the 80 participants (57 females, 23 males; mean age = 26.7, SD = 7.7), 23 reported at least one suicide attempt, while 57 had no suicide attempt history. Suicide attempters exhibited significantly higher scores on the BPI and immature defense mechanisms, particularly projection, acting out, and splitting (all *p* < 0.05). Correlation analyses revealed significant positive relationships between BPI and immature defense style (*r* = 0.55, *p* < 0.001), particularly splitting (*r* = 0.46, *p* < 0.001), projection (*r* = 0.43, *p* < 0.001), autistic fantasy (*r* = 0.41, *p* < 0.001), and acting out (*r* = 0.31, *p* < 0.001). Regression analyses were conducted using two models. The first model included age, sex, and the three defense styles (mature, neurotic, and immature) as independent variables. The second model included age, sex, and 20 individual defense mechanisms as independent variables. In the first model, immature defenses (OR = 1.035, *p* = 0.014) and female sex (OR = 4.968, *p* = 0.032) were significantly associated with history of suicide attempt. In the second model, the projection defense mechanism (OR = 1.224, *p* = 0.006) and female sex (OR = 4.071, *p* = 0.048) were significantly associated.

**Conclusion:**

These findings emphasize the importance of specific immature defense mechanisms, such as projection, and female sex, in understanding suicide attempts in individuals with BPO. Future research should investigate whether therapeutic modification of these defenses may reduce suicidality and improve outcomes.

## 1 Introduction

Borderline personality organization (BPO) represents a dimensional model of personality functioning conceptualized by Kernberg (1984). BPO represents a dimensional model of personality functioning, whereas Borderline Personality Disorder (BPD) is a categorical diagnosis defined in the *Diagnostic and Statistical Manual of Mental Disorders, Fifth Edition (DSM-5)* (American Psychiatric Association, 2013). Maintaining this distinction is important, as BPO describes a level of personality organization across a continuum, while BPD refers to a specific clinical syndrome. This model offers a structured framework for clinicians to understand personality functioning across the normal-pathological spectrum. Kernberg's approach differs from categorical models such as the *DSM-5* in that it evaluates personality functioning along a spectrum. This perspective provides a more dimensional understanding of personality structures. BPO lies between neurotic and psychotic levels and is characterized by identity diffusion, impaired reality testing under stress, ego weakness, and the predominance of maladaptive defense mechanisms (Kernberg, 1984; Caligor et al., 2018).

In coping with the stresses of daily life, individuals unconsciously utilize various psychological processes to manage internal conflicts and external stressors. Over time, various views have been put forward on the number and clustering of defense mechanisms. However, a common idea shared by psychoanalytic theorists to some degree is that the defenses are ordered on a hierarchical and continuous range mainly differentiating in the level of maturity (Vaillant et al., 1986; Perry, 1990; Cramer, 1991). Three primary defense styles are identified in a hierarchical construct: neurotic, mature, and immature (primitive). Borderline organization often characterized by the more frequent utilization of immature defense mechanisms such as splitting. These defense mechanisms play a central role in regulating affect and managing internal conflicts. Increased tendency to use immature defenses and decreased use of mature defenses are associated with impaired functioning and poor interpersonal outcomes (Granieri et al., 2017). For example, splitting, a central defense in BPO, involves perceiving people and situations in dichotomous ways, such as purely good or purely bad, which disrupts stable relationships and self-image (Kernberg, 1967, 1975). Similarly, projection involves reflecting unacceptable thoughts or feelings onto another person, allowing the individual to avoid feelings of guilt or distress, and expressing internal conflicts through impulsive and often self-destructive behaviors (Kernberg, 1992).

Individuals with BPO demonstrate some of the highest rates of suicidal behaviors among psychiatric populations, necessitating a deeper understanding of the underlying risk factors (Kjær et al., 2020; Paris, 2019; Temes et al., 2019). One study reported that nearly all individuals with borderline personality engage in self-harming behavior, 75% have attempted suicide, and the lifetime mortality rate ranges from 3 to 10% (Goodman et al., 2017). This high risk has been associated with various factors and combinations thereof, such as emotional instability, impulsivity, accompanying psychiatric disorders, and maladaptive coping strategies (Soloff et al., 2019; Links et al., 2021). While some epidemiological data show that suicide deaths are disproportionately higher among men, suicide attempts occur more frequently among women with BPO (Bozzay et al., 2014; Jeon et al., 2010; Mun et al., 2011; Lee et al., 2020; Sher et al., 2019). In a cohort study on suicide rates in the US population, the most significant predictive factor was a diagnosis of borderline personality disorder (Machado et al., 2022). Additionally, borderline personality disorder has been recognized as an independent risk factor for suicide, highlighting the internal vulnerabilities associated with this condition beyond depressive symptoms (Ando et al., 2013; Castellví et al., 2017). Moreover, BPO patients who attempt suicide show an increase in psychiatric symptoms in many areas, including depression, anxiety, substance use, aggression, hostility, and paranoid thoughts (Choi et al., 2017; Paris, 2019; Lee et al., 2020; Yang et al., 2022).

Although comorbidities such as depression and anxiety increase the risk of suicide (Paris, 2019; Yen et al., 2021), the specific contribution of defense mechanisms to suicidal tendencies in BPO has received relatively little empirical attention. We emphasized splitting and projection because these defenses, by distorting interpersonal perception and impairing reality testing, can exacerbate emotional instability and conflict escalation, thereby increasing vulnerability to suicidal crises in individuals with borderline organization. Although the above-mentioned previous studies have described the prevalence of immature defense mechanisms in individuals with BPO, very few studies have directly examined their specific relationship with suicidal behavior. Our study builds on this literature by focusing on immature defense mechanisms—particularly splitting, which fosters rigid dichotomous thinking, and projection, which externalizes blame—as potential mechanisms that may help explain how BPO pathology contributes to suicidal attempts. Recent studies incorporating network analysis approaches have further emphasized the central role of immature defense mechanisms in BPO and demonstrated strong associations with personality organization (Yun et al., 2024). Lee et al. (2020) reported that BPO patients with a history of suicide attempts used splitting, projection, and affiliation defense mechanisms more frequently than those without suicide attempts. These findings suggest that defense mechanisms may contribute to the relationship between BPO pathology and self-harming behaviors. Importantly, therapeutic approaches such as dialectical behavior therapy specifically target maladaptive defense mechanisms as a way to reduce chronic self-harm and suicidal tendencies (Yeomans et al., 2015; Euler et al., 2025). Identifying specific defense mechanisms associated with suicidal tendencies in BPO could help clinicians design interventions that directly target maladaptive coping strategies and reduce suicide attempts.

This study aims to fill an important gap in the literature by evaluating the role of defense styles in the relationship between BPO and suicide attempts, providing insights into how specific defense mechanisms contribute to suicidal behavior. We hypothesized that individuals with BPO who had attempted suicide would demonstrate significantly higher levels of spesific immature defense mechanisms, particularly splitting and projection, compared to those without such a history. We selected splitting and projection because prior research has consistently linked them to unstable mood, interpersonal difficulties, and impulsive behavior—core features of BPO that are associated with increased suicide risk.

## 2 Materials and methods

### 2.1 Participants

Participants were recruited using convenience sampling from individuals referred to the Outpatient Psychotherapy Unit of the Department of Psychiatry, Istanbul University, Faculty of Medicine, during the study period. Inclusion criteria were, age 18 years or older, and defined borderline personality organization after clinical assessment. Exclusion criteria included severe psychiatric disorders such as psychotic disorders, bipolar disorder, or intellectual disability, as well as acute medical conditions that could affect mental status or hinder participation. These criteria were applied to minimize confounding factors that could independently influence defense mechanisms and suicide attempts, thereby obtaining a more homogeneous BPO sample.

As this is a tertiary care setting specializing in psychotherapy, the sample represents treatment-seeking individuals with BPO and may not be representative of the broader BPO population in the community. During recruitment, six individuals were excluded because they did not complete or fully answer the self-report questionnaires. Only participants with complete data on all study variables were included in the analyses, resulting in a final sample of 80 participants who met all inclusion criteria.

In planning our analyses, we referred to prior research on borderline personality populations, including Lee et al. (2020), which reported a suicide attempt prevalence of 33.6% in a comparable clinical sample. While Lee et al. observed significant predictors ranging from small (OR ≈ 1.16) to large effects (OR ≈ 3.80), detecting the smallest effects would require a substantially larger sample size. Given the practical constraints of recruiting a well-characterized clinical sample, we conducted a power analysis using G^*^Power 3.1 for a binary logistic regression (α = 0.05, two-tailed, power = 0.80). With *n* = 80, the study is sufficiently powered to detect medium-to-large effects (e.g., OR ≈ 2.25), but has limited power for detecting small per-unit effects.

### 2.2 Procedures

Upon admission to unit, a psychiatry resident conducted initial assessments using a DSM-5-based semi-structured interview (American Psychiatric Association, 2013), in which diagnoses of personality disorders were established according to DSM-5 diagnostic criteria for personality disorders. These evaluations explored participants' presenting complaints and included at least three sessions for comprehensive assessment. Each clinical interview lasted ~45 min and was conducted under the supervision of experienced psychiatric residents to ensure consistency and reliability. Detailed psychiatric and developmental histories were obtained during these sessions. Subsequently, participants were evaluated by the senior professor. Concurrently, a psychologist from the research team administered the Rorschach Inkblot Test. All findings were reviewed during weekly supervision meetings attended by the supervising professor, psychiatry residents, and the psychologist. The final psychodynamic formulations and personality organization levels were determined during these meetings.

Written informed consent was obtained from all participants. Participants completed several assessments, including a Sociodemographic Data Form, Borderline Personality Inventory (BPI), Defense Style Questionnaire (DSQ-40), and Symptom Checklist-90-Revised (SCL-90-R), which were administered at the end of the first interview. All completed questionnaires were systematically reviewed for inconsistent or patterned responses.

The study was approved by the Ethics Committee of Istanbul University, Istanbul Faculty of Medicine (Approval No.: 2498493, Date: March 26, 2024), and all participants provided signed informed consent.

### 2.3 Measurements

#### 2.3.1 Sociodemographic data form

This form, developed by the researchers, was used to collect detailed sociodemographic and clinical information, including age, sex, education level, marital status, employment status, history of suicide attempts, psychiatric treatment history, and use of alcohol, substance, and tobacco. The purpose of this form was to gather comprehensive background information to contextualize the study findings and identify potential confounding variables.

#### 2.3.2 Borderline Personality Inventory (BPI)

The BPI, a self-report instrument, based on Kernberg's structural personality organization theory, consists of 53 true-false items designed to assess the level of BPO. The final two items are used only for clinical detection, while the first 51 items are included in the total score analysis. Each item is scored 1 for “true” and 0 for “false,” with total scores ranging from 0 to 51. The BPI has demonstrated good psychometric properties in both clinical and non-clinical populations (Leichsenring, 1999). In Turkish, the Cronbach's alpha value for the reliability analysis of the entire study group was determined as 0.92, while that for the borderline personality disorder group was determined as 0.84. A cut-off score of 15/16 is generally recommended to differentiate individuals with significant borderline features in Turkish sample (Aydemir et al., 2006).

#### 2.3.3 Defense Style Questionnaire (DSQ-40)

The DSQ-40 is a self-report scale consisting of 40 items rated on a 9-point Likert scale, assessing 20 defense mechanisms classified into three styles: mature (Sublimation, Humor, Suppression, Anticipation), neurotic (Undoing, Idealization, Pseudo Altruism, Reaction Formation), and immature (Rationalization, Projection, Denial, Devaluation, Acting Out, Somatization, Autistic Fantasy, Splitting, Passive Aggression, Displacement, Isolation, Dissociation) (Andrews et al., 1989). The Turkish version of the scale has been validated and shown to have acceptable psychometric properties (Yilmaz et al., 2007). Higher mean scores reflect greater use of specific defense mechanisms. In this study, the DSQ-40 was used to identify predominant defense styles and examine their relationship with suicide attempts in individuals with BPO.

#### 2.3.4 Symptom Checklist-90-Revised (SCL-90-R)

The SCL-90-R is a 90-item multidimensional questionnaire assessing a broad range of psychological symptoms and distress (Derogatis et al., 1976). Items are rated on a 0–4 Likert scale, forming subscales for somatization, obsessive-compulsive symptoms, interpersonal sensitivity, depression, anxiety, hostility, phobic anxiety, paranoid ideation, and psychoticism. In this study, the SCL-90-R was used to assess overall psychiatric symptom burden and specific symptom dimensions, providing a detailed profile of participants' psychological distress. The Turkish adaptation and validation of the SCL-90-R have demonstrated acceptable psychometric properties, supporting its reliability and suitability for use in clinical and research settings (Dag, 1991).

### 2.4 Statistical analyses

The IBM Statistical Package for the Social Sciences (SPSS) 28th edition was used for data analysis. *p*-values below 0.05 were considered as statistically significant in all analyses. The participants' descriptive statistics are reported as means, standard deviations, medians, and percentages. Non-parametric tests were selected due to the non-normal distribution of key variables, as assessed by the Shapiro–Wilk test. The Mann–Whitney *U*-test was used for continuous variable comparisons, whereas the Chi-square was employed for categorical variable comparisons. Fisher's Exact Test was used because of small cell counts. Spearman's correlation analysis was conducted to examine the relationships between numerical variables. The effect of various variables on suicide attempt history were analyzed using binomial logistic regression with both stepwise and enter methods, in which suicide attempt history was considered the dependent variable. All defense styles or defense mechanisms, age, and sex were used as independent variables in these analyses. Prior to the regression analyses, multicollinearity among independent variables was assessed using Variance Inflation Factor (VIF). All VIF values ranged between 1.00 and 2.50, indicating no significant multicollinearity. Model fit was evaluated using the Hosmer-Lemeshow goodness-of-fit test (*p* >0.05) and Nagelkerke *R*^2^ values (*p* < 0.001), both of which indicated acceptable model performance.

## 3 Results

### 3.1 Socio-demographic characteristics

A total of 80 participants with BPO (57 females, 23 males) were included in the study, with a mean age of 26.7 years (SD = 7.7). Of these, 23 participants (28.8%) reported at least one histoy of lifetime suicide attempt, while 57 reported no such history. There were no statistically significant differences between the suicide attempter and non-attempter groups according to sex, age, educational level, marital status, employment status, psychiatric treatment history, or alcohol, substance, and tobacco use (Table 1).

**Table 1:** Comparison of the socio-demographic and clinical characteristics of participants according to history of suicide attempt.

**Variables**	***n***	**Median (min–max)**	**Suicide attempter Median (min–max)**	**Non–suicide attempter Median (min–max)**	***U***	***p***
Age	80	23 (18–50)	26 (42–18)	23 (50–18)	−0.107	0.915
		**%**	***n*** **(%)**	***n*** **(%)**	*χ^2^*	***p***
Participants	80	100	23 (28.7%)	57 (71.3%)		
**Sex**						
Female	57	71.3	20 (87%)	37 (64.9%)	3.888	0.059^+^
Male	23	28.7	3 (13%)	20 (35.1%)		
**Education**						
High school	39	48.8	10 (43.5%)	29 (50.9%)	2.757	0.378
Undergraduate	38	47.5	13 (56.5%)	25 (43.9%)		
Graduate	3	3.7	0 (00.00%)	3 (5.2%)		
**Working status**						
Not-working/unemployed	13	16.3	4 (17.4%)	9 (15.8%)	1.538	0.459
Working	24	30.0	9 (39.1%)	15 (26.3%)		
Student	43	53.7	10 (43.5%)	33 (57.9%)		
**Marital status**						
Married	11	13.8	2 (8.7%)	9 (15.7%)	0.695	0.497^+^
Non-married	69	86.2	21 (91.3%)	48 (84.3%)		
**Psychiatric treatment history**						
No	34	43.6	10 (45.5%)	24 (42.9%)	0.043	0.835
Yes	44	56.4	12 (54.5%)	32 (57.1%)		
**Alcohol usage**						
No	36	45.0	9 (39.1%)	27 (47.4%)	2.754	0.217
Socially drink	37	46.3	10 (43.5%)	27 (47.4%)		
Previous alcohol use Disorder	7	8.7	4 (17.4%)	3 (5.2%)		
**Substance usage**						
No	69	86.3	20 (87.0%)	49 (86.0%)	0.768	0.691
A few times	6	7.4	1 (4.3%)	5 (8.8%)		
Previous substance use disorder	5	6.3	2 (8.7%)	3 (5.2%)		
**Tobacco usage**						
No	46	61.3	10 (50.0%)	36 (65.5%)	2.902	0.197
Former smoker	3	4.0	2 (10.0%)	1 (1.8%)		
Active smoker	26	34.7	8 (40.0%)	18 (32.7%)		

U, Mann–Whitney U-test; χ^2^, Chi-Square test; ^+^Fisher's exact test. Values of *p* < 0.05 were accepted as significant.

### 3.2 Clinical characteristics

We found that suicide attempters had significantly higher scores on the BPI (*p* = 0.004), immature defense style (*p* = 0.016), projection (*p* = 0.008), acting out (*p* = 0.045), and splitting (*p* = 0.026) defense mechanism scores than non-suicide attempters (Table 2). Additionally, total scores and most subscale scores of the SCL-90-R were significantly higher among suicide attempters (all *p* < 0.05), with the exception of interpersonal sensitivity and psychoticism (*p* = 0.335 and *p* = 0.526, respectively; Table 3).

**Table 2:** Comparison of the borderline personality level, defense styles, and all defense mechanisms according to suicide attempt.

**Variables**	**Total Median (min–max) (*n* = 80)**	**Suicide attempter Median (min–max) (*n* = 23)**	**Non-suicide attempter Median (min–max) (*n* = 57)**	***U***	***p***
Borderline personality inventory	21 (4–39)	25 (7–39)	19 (4–33)	−2.872	**0.004**
**Defense style questionnaire**					
Mature defense style	38 (10–64)	41 (10–59)	37 (15–64)	−0.542	0.588
1. Sublimation	7.5 (2–18)	7 (2–18)	8 (2–18)	−0.421	0.674
2. Humor	9.5 (2–18)	10 (2–16)	9 (2–18)	−0.261	0.794
3. Suppression	7.5 (2–18)	8 (3–18)	7 (2–18)	−1.212	0.225
4. Anticipation	11 (2–18)	10 (2–18)	11 (2–18)	−0.021	0.983
Neurotic defense style	38 (21–59)	36 (13–54)	39 (21–59)	−0.857	0.392
1. Undoing	9 (2–18)	10 (2–18)	8 (2–18)	−1.498	0.831
2. Idealization	8 (2–18)	7 (2–16)	9 (2–18)	−0.839	0.582
3. Pseudo altruism	11.5 (3–18)	10 (3–18)	12 (4–18)	−0.929	0.169
4. Reaction formation	10 (2–18)	10 (2–18)	10 (2–17)	−0.688	0.264
**Immature defense style**	100 (38–145)	109 (57–145)	97 (38–144)	−2.808	**0.016**
1. Rationalization	10 (2–16)	10 (4–15)	10 (2–16)	−1.264	0.640
**2. Projection**	11 (2–18)	14 (6–18)	11 (2–18)	−3.182	**0.008**
3. Denial	6 (2–18)	7 (2–13)	6 (2–18)	−0.182	0.814
4. Devaluation	7 (1–16)	7 (2–16)	6 (1–16)	−0.738	0.777
**5. Acting out**	10 (2–18)	11 (2–18)	9 (2–18)	−1.849	**0.045**
6. Somatization	10 (2–18)	9 (2–17)	10 (2–18)	−0.990	0.400
7. Autistic fantasy	10 (2–18)	10 (2–18)	9 (2–18)	−1.614	0.154
**8. Splitting**	9 (2–18)	10 (2–18)	9 (2–18)	−2.639	**0.026**
9. Passive aggression	8 (2–18)	10 (2–18)	8 (2–18)	−1.637	0.303
10. Displacement	9 (2–18)	9 (2–18)	9 (2–15)	−1.176	0.308
11. Isolation	8 (2–18)	9 (2–18)	7 (2–18)	−0.909	0.118
12. Dissociation	4 (2–15)	5 (2–15)	4 (2–14)	−0.479	0.425

U, Mann–Whitney U-test, Values of *p* < 0.05 were accepted as significant and marked in bold.

**Table 3:** Comparison of the symptom checlist-90 total and subscale scores according to suicide attempt.

**Variables**	**Total Median (min–max) (*n* = 80)**	**Suicide attempter Median (min–max) (*n* = 23)**	**Non-suicide attempter Median (min–max) (*n* = 57)**	***U***	***p***
**SCL-total**	1.8 (0.3–3.2)	2.1 (0.3–3.2)	1.5 (0.5–3.2)	−3.054	**0.002**
**Somatization**	1.3 (0.2–4)	1.6 (0.2–4)	1.1 (0–3.3)	−2.431	**0.015**
**Obsessive-compulsive**	2.2 (0.5–3.8)	2.5 (0.9–3.7)	2 (0.5–3.8)	−2.474	**0.013**
Interpersonal sensitivity	2.1 (0.1–3.8)	2.2 (0.1–3.8)	2 (0.3–3.4)	−0.964	0.335
**Depression**	2.5 (0.6–3.6)	2.9 (1.2–3.5)	2.4 (0.6–3.6)	−2.547	**0.011**
**Anxiety**	1.5 (0–4)	1.9 (0–3.6)	1.3 (0.1–4)	−2.584	**0.010**
**Hostility**	1.7 (0–4)	2.3 (0–4)	1.3 (0–3.7)	−2.896	**0.004**
**Phobic anxiety**	0.7 (0–3.4)	1.4 (0–3.4)	0.6 (0–3.4)	−2.393	**0.017**
**Paranoid ideation**	1.8 (0–3.8)	2.3 (0–3.8)	1.7 (0.2–3.3)	−2.808	**0.005**
Psychoticism	1 (0.1–2.9)	1.1 (0.1–2.9)	1 (0.1–2.7)	−0.633	0.526
**SCL-others**	1.9 (0.3–3.6)	2.3 (0.6–3.6)	1.5 (0.3–3.0)	−3.325	**<** **0.001**

U, Mann–Whitney U-test, Values of *p* < 0.05 were accepted as significant and marked in bold.

### 3.3 Correlation analysis of borderline personality features with age, defense styles and defense mechanisms

Correlation analyses revealed a significant negative association between BPI scores and age (*r* = −0.27, *p* < 0.05), and a significant positive correlation with the immature defense style (*r* = 0.55, *p* < 0.001). Significant positive correlations were also observed between BPI scores and specific immature defense mechanisms, including splitting, projection, autistic fantasy, acting out, devaluation, displacement, passive aggression, and denial (all *p* < 0.05). No significant associations were found with rationalization, isolation, somatization, or dissociation (Table 4).

**Table 4:** Correlation analysis of borderline personality features and defense mechanisms (*n* = 80).

**Variables**	**Borderline personality inventory (*r*)^+^**
**Age**	**−0.27** ^*^
**Immature defense style**	**0.55** ^**^
Rationalization	0.16
**Projection**	**0.43** ^**^
**Denial**	**0.23** ^*^
**Devaluation**	**0.29** ^*^
**Acting out**	**0.31** ^*^
Somatization	0.15
**Autistic fantasy**	**0.41** ^**^
**Splitting**	**0.46** ^**^
**Passive-aggression**	**0.26** ^*^
**Displacement**	**0.28** ^*^
Isolation	0.13
Dissociation	0.19
Neurotic defense style	0.11
Pseudo-altruism	0.01
Reaction formation	0.02
Idealization	0.13
Undoing	0.13
Mature defense style	0.12
Suppression	0.22
Sublimation	0.07
Humor	0.03
Anticipation	0.03

^+^Spearman correlation analysis.

^*^p < 0.05.

^**^p < 0.001.

Values of *p* < 0.05 were accepted as significant and marked in bold.

### 3.4 Regression analyses of suicide attempt according to sex, defense styles and defense mechanisms

In the first regression model, binominal logistic regression analysis was conducted with suicide attempt history as the dependent variable and sex, age, and the three defense styles as independent variables. Immature defense style [OR = 1.035, 95% CI (1.007, 1.065), *p* = 0.014] and female sex [OR = 4.968, 95% CI (1.149, 21.487), *p* = 0.032] were significantly associated with suicide attempt history. Each one-unit increase in immature defense style score was associated with a 3.5% increase in the odds of a suicide attempt. Female participants had nearly five times greater odds of having a history of suicide attempt compared to males (Table 5a).

**Table 5:** Logistic regression analyses (a, b) (*n* = 80).

**Dependent variable**	**Suicide attempt**					
**a. Independent variables (enter method)**	**Beta**	**SE**	***p***	**OR**	**%95 CI of OR**	
					**Lower**	**Upper**
Constant	−0.131	2.248	0.954	0.036		
**Sex**	−1.603	0.747	**0.032**	4.968	1.149	21.487
Age	0.001	0.037	0.984	1.001	0.931	1.076
Mature defense style	−0.004	0.023	0.853	0.996	0.951	1.042
Neurotic defense style	−0.060	0.031	0.057	0.942	0.886	1.002
**Immature defense style**	0.035	0.014	**0.014**	1.035	1.007	1.065
**b. Independent variables**						
**(forward stepwise method)**						
Model 1						
Constant	−3.188	0.926	0.001	0.041		
Projection	0.189	0.070	0.007	1.208	1.053	1.386
Model 2						
Constant	−1.623	1.191	0.173	0.012		
**Sex**	−1.404	0.711	**0.048**	4.071	1.009	16.415
**Projection**	0.202	0.740	**0.006**	1.224	1.059	1.414

Dependent: variable: history of suicide attempt (yes/no).

a. Independent variables: sex, age, mature defense style, neurotic defense style, immature defense style.

Model 1: Nagelkerke R^2^ = 0.229 (*p* < 0.05).

b. Independent variables: sex, age, all defense mechanisms.

Model 1: Nagelkerke R^2^ = 0.143 (*p* < 0.05).

Model 2: Nagelkerke R^2^ = 0.216 (*p* < 0.05).

SE, standard error; OR, odds ratio; CI, confidence interval.

Values of *p* < 0.05 were accepted as significant and marked in bold.

In a second regression model, binominal logistic regression analysis was conducted with suicide attempt history as the dependent variable and sex, age, and 20 individual defense mechanisms as independent variables. Projection [OR = 1.224, 95% CI (1.059, 1.414), *p* = 0.006] and female sex [OR = 4.071, 95% CI (1.009, 16.415), *p* = 0.048] remained significant predictors. Frequent use of projection was associated with a 22.4% increase in the odds of having attempted suicide. Female participants had 4.07 times higher odds of reporting a history suicide attempt (Table 5b).

## 4 Discussion

This study investigated the relationships between defense mechanisms and suicide attempts in individuals with BPO. Approximately one-third of participants reported a history of suicide attempt, a finding consistent with earlier reports estimating that up-to 85% of individuals with BPO attempt suicide during their lifetime (Paris, 2019; Oumaya et al., 2008; Soloff et al., 2019; Links et al., 2021). Such variability in reported rates may stem from methodological differences, sample characteristics, and diagnostic criteria.

Our findings did not reveal significant sociodemographic differences between individuals with and without history of suicide attempt. This result is largely consistent with previous findings (Lee et al., 2020), although some studies have reported higher suicide attempt rates among females with BPO (Jeon et al., 2010; Mun et al., 2011; Bozzay et al., 2014). Conversely, a study reported that suicide mortality rates appear to be higher among males than among female with BPO (Sher et al., 2019). In clinical samples, females were diagnosed with BPO more frequently and are more likely to be admitted to treatment than males; however, findings from non-clinical samples suggest that BPO prevalence is similar in both sexes (Zanarini et al., 2011). Notably, regression analysis revealed female sex as a significant contributor of suicide attempt history, highlighting the need for sex-sensitive risk assessments in this population. Although males have been found to have higher suicide mortality rates, females with BPO may present with more frequent suicide attempts and should receive tailored clinical attention.

Regarding psychopathology, participants with suicide attempts exhibited significantly higher SCL-90-R total and subscale scores—including depression, anxiety, hostility, and paranoid ideation—compared to non-attempters. These findings are consistent with previous literature showing that BPO individuals with suicidal behaviors experience elevated psychiatric symptom burden (Choi et al., 2017; Lee et al., 2020; Yang et al., 2022). Of particular note, hostility and aggressive tendencies that symptoms linked to immature defenses, were prominent, supporting Kernberg's conceptualization of BPO as a polysymptomatic construct characterized by affective instability, poor impulse control, and comorbid psychopathology. Individuals with BPO who attempt suicide exhibit a broader range of psychiatric symptoms and disorders, emphasizing the complexity and multifaceted nature of suicidal behavior in this population. The absence of significant differences in interpersonal sensitivity and psychoticism between groups may reflect that these dimensions are less central to suicide risk within BPO populations, or that our sample size limited the ability to detect smaller effects. Our data also indicated significantly higher BPI scores among suicide attempters, suggesting that individuals with more pronounced borderline features may be more prone to suicidal behaviors.

We also found a strong association between immature defense style and borderline personality features, with splitting emerging as the most prominent mechanism. This aligns with Kernberg's theoretical model, which posits that individuals with BPO rely heavily on maladaptive defenses—particularly splitting and other primitive mechanisms such as projection and acting out (Kernberg, 1967, 1975). While suppression is considered characteristic of neurotic-level personality organization, splitting is central to BPO, reflecting a failure to integrate contradictory emotional experiences. Our findings are consistent with recent network-analytic research, which demonstrated that immature defense mechanisms occupy a central role in the personality architecture of BPO, showing strong associations with schizotypal, dependent, and narcissistic traits (Yun et al., 2024). These findings contribute to our understanding of how maladaptive defense mechanisms, such as splitting, are intricately linked to elevated suicide risk in individuals with BPO. Clinically, this suggests that therapeutic interventions should not only address impulsivity and affect regulation, but also focus on modifying maladaptive defenses, potentially through psychodynamic approaches. A significant negative correlation was also observed between age and borderline features, which is consistent with prior research showing that individuals with neurotic-level personality organization tend to be older and exhibit lower suicide risk (Sahin et al., 2024). This developmental pattern may reflect a maturation of defensive functioning over time, with a gradual shift away from primitive coping strategies.

Regression analyses in our study revealed that immature defense style, particularly the projection mechanism, along with female sex, was significantly associated with a history of suicide attempts. Projection is characterized by attributing one's own unacceptable impulses to others. In BPO, it serves to externalize negative internal representations and may reduce current distress. However, its long-term use has been associated with self-esteem impairment and the accumulation of internalized anger, and may contribute to suicidal behavior (Kernberg, 1992). These findings are consistent with previous research that explored relationship between personality organization and the immature defense mechanisms and showed that immature defenses associated with greater borderline personality features and pathological personality traits (Cramer, 1999; Lingiardi et al., 1999; Shafiee Tabar, 2018; Carvalho and Pianowski, 2019). Similarly, a study showed that immature defense styles may be associated with higher suicide attempt in patients with BPO. They reported that in BPO patients with suicide attempt history, there was a substantially tended to utilize more frequently splitting, projective identification, and affiliation defense mechanisms compared to the non-suicide attempter group (Lee et al., 2020). Additionally, according to two interview-based old studies, immature defenses are related to the borderline organization, aggression, and impulsiveness (Perry and Cooper, 1989; Bond, 1990). These findings suggest that immature defense style may indicate aggression and immature defense mechanisms suicide attempt in patients with BPO. Findings in present study suggest that immature defense style, projection, and female sex may serve as clinically relevant markers for identifying individuals with BPO at elevated suicide risk, potentially informing targeted risk assessment and intervention strategies. Psychotherapeutic approaches such as dialectical behavior therapy and transference-focused psychotherapy explicitly target maladaptive defenses in individuals with BPO, aiming to reduce chronic suicidality and self-injury (Yeomans et al., 2015; Euler et al., 2025). Moreover, long-term dynamic psychotherapy has been shown to foster adaptive defensive restructuring, with changes in defense functioning mediating symptomatic and functional improvements (Perry and Bond, 2012). These insights emphasize the importance of integrating defense-oriented strategies into clinical interventions for high-risk BPO populations.

This study has several limitations that should be considered when interpreting the findings. First, it was a cross-sectional study conducted in a university hospital (a tertiary care center), which limits the generalizability of the results to all individuals with BPO, particularly those in community or primary care settings, and precludes causal inferences. While a causal relationship cannot be established, the observed associations suggest that the use of immature defense mechanisms may increase emotional instability and interpersonal conflicts, thereby elevating suicide risk. Future studies employing larger, multi-site, and longitudinal designs are needed to clarify the temporal and causal relationships between defense mechanisms and suicidality.

Second, the small number of participants and individuals with a history of suicide attempt may have reduced statistical power for detecting weaker associations, affecting the robustness of subgroup analyses. Although the power analysis indicated sufficient power to detect medium-to-large effects (e.g., OR ≈ 2.25) with *n* = 80, the study has limited power for detecting small per-unit effects such as those observed in prior studies (e.g., OR ≈ 1.16 in Lee et al., 2020). This limitation should be considered when interpreting non-significant findings for weaker predictors.

Third, although patients underwent a thorough assessment, all psychological characteristics were measured using self-report scales (BPI, DSQ-40, SCL-90-R), which are subject to response bias. This risk may be particularly relevant in individuals with BPO, who can have difficulties with self-perception and insight. To mitigate this, questionnaires were checked for inconsistent or random responses, and follow-up clinical interviews were used when necessary; however, residual bias cannot be excluded.

Fourth, although sociodemographic and clinical data were collected, potentially important confounding variables—such as medication status, illness duration, and psychiatric comorbidities—were not controlled for in the analyses. These factors may influence both defense mechanisms and suicide risk, and their omission may have biased the findings.

Finally, the gender differences observed—particularly the higher odds of suicide attempts among women—were not examined in depth in relation to sociocultural or clinical factors. Future research should explore these dimensions to better understand the underlying mechanisms and to inform gender-sensitive suicide prevention strategies.

In conclusion, this study emphasizes the important role of immature defense mechanisms, particularly projection, in relation to suicide attempts in individuals with BPO. Female sex also appears to be an important contributing factor. These findings suggest that immature defense mechanisms may contribute to self-harming behavior in this population and should be assessed in clinical settings. Interventions focused on defense mechanisms, particularly those supported by empirical evidence, may offer encouraging perspectives for suicide prevention.

## Data Availability

The raw data supporting the conclusions of this article will be made available by the authors, without undue reservation.
